# Straightlining prevalence across domains of social media use and impact on internal consistency and mental health associations in the LifeOnSoMe study

**DOI:** 10.1038/s41598-025-14276-6

**Published:** 2025-08-07

**Authors:** Jens Christoffer Skogen, Amanda Iselin Olesen Andersen, Gunnhild Johnsen Hjetland, Leif Edvard Aarø, Ian Colman, Børge Sivertsen, Turi Reiten Finserås

**Affiliations:** 1 Department of Health Promotion, Norwegian Institute of Public Health, Bergen, Norway; 2 Centre for Evaluation of Public Health Measures, Norwegian Institute of Public Health, Oslo, Norway; 3 Alcohol and Drug Research Western Norway, Stavanger University Hospital, Stavanger, Norway; 4 School of Epidemiology and Public Health, University of Ottawa, Ottawa, Canada; 5 Centre for Fertility and Health, Norwegian Institute of Public Health, Oslo, Norway; 6 Department of Research and Innovation, Helse Fonna HF, Haugesund, Norway

**Keywords:** Psychology and behaviour, Epidemiology, Population screening

## Abstract

Straightlining (uniform responses across items), poses a risk in surveys. Among adolescents, previous studies have investigated the prevalence and impact of straightlining in shorter questionnaires within larger surveys. A typical finding is that straightlining is more common among younger respondents, and particularly among boys. A better understanding of straightlining is important for improving data quality. The present study aims to estimate the prevalence of straightlining among adolescents filling out a survey covering different aspects of social media use across 64 items. Additionally, it seeks to assess the impact of straightlining on internal consistency and the associations between six domains of social media use and symptoms of anxiety and depression. Data from the «LifeOnSoMe»-study (*N* = 3,285), collected from adolescents (aged 16+) in Bergen, Norway. Descriptive and inferential statistics. In total, 5.4% of participants were straightliners, (8.6% of the boys vs. 2.9% of the girls (*p* < 0.001)). There were no differences in age between straightliners and the remainder of the sample. Overall, the prevalence and impact of straightlining was limited in the present sample. However, there were large discrepancies in terms of both internal consistency, correlations between domains of social media use, and associations with symptoms of anxiety and depression between straightliners and the remainder of the sample. Straightlining behavior had minimal effects on this sample’s analytical epidemiological conclusions. While boys were more prone to straightlining than girls, overall prevalence was low. However, significant discrepancies between straightliners and other respondents suggest potential risks in samples with higher straightlining prevalence.

**Supplementary Information:**

The online version contains supplementary material available at 10.1038/s41598-025-14276-6.

## Background

 Population-based surveys rely on the honest and attentive self-report of participants to yield accurate insights^1^. Careless responding is a potential threat to both the reliability and validity of measurements^1^. In terms of reliability, careless responding can lead to a lack of consistency and estimates of reliability can be artificially lowered or inflated. Furthermore, validity can be compromised, which may lead to a systematic bias in the findings and the subsequent interpretations of these. A special case of carelessness in responding is the tendency of participants to give the same response to a series of questions (i.e., non-differentiation), often called ‘straightlining’^1^. Perfect straightlining is defined as the exact same answer to all questions in a series, while partial straightlining indicates a pattern of unusually identical responses to consecutive questions in a series. As the number of questions in a series increases, perfect straightlining is less likely and partial straightlining may be a better indicator of careless responding^2^.

It is important to note that some degree of similarity in responses can be valid, depending on the nature of the questions and the respondent’s true attitudes or experiences. For instance, we expect that questions related to the same underlying construct would elicit similar responses from the respondent. The distinction between valid and invalid straightlining is therefore important. Invalid straightlining is thought of as being an indication of low-effort responding, and may be particularly prevalent in longer or more demanding surveys, where respondents might be prone to cognitive fatigue and reduced motivation^3^. Straightlining behaviors are associated with the *satisficing* theory, which posits that when respondents experience high cognitive demands or low motivation, they may shift to response strategies that reduce the mental effort required to answer questions accurately. In such cases, respondents may engage in weak satisficing, where they provide responses that are acceptable but less thoughtful, for example, by choosing the first plausible option. Alternatively, respondents may engage in strong satisficing, where they bypass deeper cognitive processing and adopt response patterns like straightlining by selecting the same answer repeatedly without adequally considering the content of each question^3^. Studies indicate that satisficing is relatively common, with its prevalence increasing as task difficulty rises (e.g., lengthy questionnaires or complex question formats), respondent ability decreases (e.g., lower educational levels or lack of domain-relevant knowledge), and respondent motivation wanes (e.g., survey fatigue or reluctance to participate)^4^.

Satisficing strategies, particularly straightlining, have been more commonly observed among younger respondents, potentially because of factors like higher susceptibility to survey fatigue, tendencies towards shortcuts, and a higher likelihood of impatience or low engagement, especially in long or repetitive surveys^5,6^. Additionally, lower conscientiousness may also contribute to a prioritization of speed over precision as cognitive fatigue sets in^7^. Previous studies have also found gender differences among younger respondents, and straightlining seems to be far more common among boys compared to girls^8^. The difference in straightlining between boys and girls can potentially be attributed to several factors. Research suggests gender differences in engagement levels and motivations when participating in surveys^9^. Boys might find surveys less engaging or more tedious, leading to a higher likelihood of straightlining as a way to complete quickly^6^. Cognitive and behavioral differences between genders, such as attention span and response styles^10^, could also play a role. Additionally, broader characteristics such as findings indicating generally higher levels of conscientiousness^11^ and prosocial behaviour^12^ among girls compared boys can potentially also be part of the gender difference in straightlining.

Understanding the prevalence and impact of straightlining is crucial, as straightlining will typically inflate reliability estimates such as Cronbach’s alpha and may artificially influence (weaken or strengthen) the observed associations between the items being straightlined and other variables. Thus, it may compromise the findings and subsequent interpretation^13^.

In short, straightlining remains a substantial concern in surveys of adolescents, and more research is needed to understand how straightlining might impact reliability and validity. Previous studies have shown that the prevalence of straightlining differs across cross-cultural surveys of adolescents^8^. Estimating the prevalence is in itself worthwhile, but the prevalence also plays a major role with regard to the potential consequences of straightlining. By improving our knowledge about the extent and impact of this behaviour, we might be able to contribute to better survey designs and improving data quality. Specifically, understanding the prevalence of straightlining is crucial because its presence can significantly affect data quality and survey results. By identifying straightlining, researchers can mitigate or at least take into consideration the potential implication on their results^6,8^.

A particularly timely subject within mental health epidemiology in the last decades, has been on the relationship between use of social media and mental health and wellbeing among adolescents^14–16^. Most early research within this field has focused on the amount of use, but in recent years there has been a call for a more detailed and broad mapping of different aspects of social media – to better understand the potential link with mental health and wellbeing. Although some aspects of social media use may be monitored or at least gleaned from objective data – such as usage patterns across platforms and exposure to specific content – the subjective aspects of social media use still rely heavily on self-report^17^. Objective data can provide insights into how often users engage with different platforms, the types of content they interact with, and the duration of their usage. However, understanding the personal experiences, motivations, and emotional responses of users requires self-report data^18^. Mapping this complexity increases the length and depth of surveys focusing on social media use. Consequently, the detailed nature of these surveys can lead to respondent fatigue, potentially increasing the likelihood of straightlining behavior. This, in turn, could lead to poorer overall data quality and a potential misrepresentation and misinterpretation of the actual relationship between social media use and mental health and wellbeing. To the best of our knowledge, no previous studies have investigated the potential impact of straightlining in relation to social media use among adolescents.

### Aims

The aim of the present study is to estimate the prevalence of straightlining among adolescents completing a survey covering different domains of social media use with identical response options. We also wanted to investigate the impact of straightlining on six specific domains of social media use – self-presentation, negative acts and exclusion, unwanted attention, subjective overuse, social obligations, and source of concern - in terms of internal consistency and associations with symptoms of anxiety and depression.

## Methods

### Data

The present study is based on data from the school-based “LifeOnSoMe”-study, a survey investigating different aspects of adolescents’ social media use, and mental health and well-being. The target population was adolescents attending senior high school. The study took place in Bergen, Norway (2020/2021) and over 3,500 adolescents aged 16 (mean age 17.4 years) or older took part. 44% of the total sample were boys. Data collection was electronic, and participants had to give their informed consent before agreeing to take part. One school hour (minimum 45 min) was allotted to complete the questionnaire. More information about the study and recruitment is available in previous publications^19–21^. A total of 3,285 participants had valid responses to all variables of interest and were included in the analytical sample.

### Measures of social media

In a discrete section of the survey, the adolescents were presented with a range of questions and statements regarding their use, beliefs, experiences, and attitudes towards social media. In addition to a preamble with questions regarding the amount of social media use and which platforms they use, they were presented with 80 statements covering several different aspects and domains of social media use (see pages 2–4 of Supplementary material for an overview). This social media section of the survey was developed based on review-informed qualitative interviews with adolescents to make them relevant and closely connected to the target population’s lived experience with social media. Item development followed the steps indicated by Boateng and colleagues^22^, with some differences: The aspects covered in the survey emerged primarily from themes identified from the qualitative interviews with adolescents^23^, but were also informed by previous research on the subject^24,25^. Additionally, we allowed for single items covering different aspects of social media use not directly part of any of the themes (see pages 2–4 of Supplementary material for an overview).

The domains covered included:

I. Self-presentation (7 items).
II. Negative acts and exclusion (5 items).
III. Unwanted attention from others (3 items).
IV. Subjective overuse (5 items).
V. Social obligations (8 items).
VI. Source of concern (3 items).

In addition, other aspects were covered such as what the participants use social media for, digital stress related to social media, efforts related to regulation of use and digital hygiene, perceived norms on social media, and social aspects of online gaming. The full list of the 80 items is provided in the supplementary material, including color coding for the specific domains listed above (see Supplementary material, pages 2–4). The first 16 items were related to what social media were used for, while the remaining 64 items covered other aspects related to social media use. The last 64 items and the domains listed above (I-VI) will be the focus of inquiry with respect to straightlining and its potential impact on associations with outcomes (see analytical plan below for further details). The six domains were chosen as a focus in our inquiry as we have previously found that they are associated with mental health and well-being^20,21,26,27^. It is also likely that they represent particularly pertinent aspects of social media use in relation to negative potential impacts [See for instance^15,28–30^].

### Mental health measures

#### Symptoms of anxiety

Symptoms of anxiety were measured by the General Anxiety Disorder 7 questionnaire (GAD-7^31^). Seven questions regarding symptoms of general anxiety scored from 1 (not at all) to 4 (almost every day) make up the GAD-7. The questionnaire can be used as a continuous measure (total score, ranging from 0 to 21 in this study (28 maximum)) or as a dichotomous variable with a cut-off of 10 to define case-level. Cronbach’s alpha was 0.89 in the present sample. For the purposes of the present study, GAD-7 was used as a continuous variable (see statistical analysis section).

#### Symptoms of depression

Symptoms of depression were measured by the Short Mood and Feelings Questionnaire (SMFQ^32^). Thirteen statements related to symptoms of depression with the following response options 0 (not true), 1 (sometimes true), and 2 (correct) constitute the SMFQ. The questionnaire can be used as a continuous measure (total score, ranging from 0 to 26) or as a dichotomous variable with a cut-off at the 90th percentile to define case-level. Cronbach’s alpha was 0.91 in the present sample. For the purposes of the present study SMFQ was used a continuous variable (see statistical analysis section).

### Definition of straightlining

Assessment of straightlining was based on the longest string of identical consecutive responses for each respondent across 64 successive items of statements related to social media use. All statements had five potential response options: “Not at all”, “A little”, “Somewhat/partly agree”, “A lot”, and “Very much” / “Never”, “Seldom”, “Sometimes”, “Often”, and “Very often”. Straightliners were defined as statistical outliers (1.5 times the interquartile range greater than the third quartile^33^ for longest string of identical statements across the 64 items (see Supplementary material, Figure S1)). In simpler terms, if a participant gave the same answer to an unusually long consecutive string of questions compared to other respondents, they were labeled as a straightliner. The cut-off for straightlining was 16 or more consecutive identical responses. This method allows for some repetition in answers (which can be natural), but flags those with extreme patterns of repetition. This definition enables us to identify perfect and partial straightliners, which is beneficial across longer sets of items not meant to gauge the same underlying construct [See for instance: 2]. It should be noted that our definition of straightlining means that the maximum prevalence possible would be 24.9% of the total sample.

### Analytical plan

After identification of straightliners based on the definition provided above, descriptive statistics of gender, age, total time to complete the survey and the longest string of identical responses on the social media responses were calculated for the overall sample, as well as separately for straightliners and the remainder of the sample. For gender and age, the frequency and proportions were estimated, and for time to complete and the longest string of identical responses the median and interquartile range (Q1, Q3) were estimated as well as the 5% and 95% percentile for the latter variable.

Differences between straightliners and the remainders were estimated using Pearson’s Chi-squared tests for categorical variables, while Wilcoxon rank sum tests were used for continuous measures. The mean and standard deviation across straightliners and the remainder of the sample and the response distribution for each social media item was also computed and presented in the Supplementary material (see Table S1, and figures S2 and S3). Then the response patterns across the 64 social media items were investigated among straightliners versus the remainder of the sample. To do this, the most frequent response (mode response) across the items for each individual was estimated, and this response was then subtracted from each of the items, yielding a potential deviation score of +/- 4 for each item, where 0 indicates no deviation from their most frequent response. These response patterns were then visually presented as two separate heatmaps, one for the straightliners and one for the remainder of the sample. To compare the deviation from the participants’ typical responses among straightliners versus the remainder, the average absolute deviation across the 64 items were computed, and the effect size for the comparison was estimated using the robust effect size index presented with 95% confidence intervals (95%CI) based on 1,000 non-parametric bootstraps^34^. Next, the reliability of the six social media domains of interest (I-VI) was estimated using Cronbach’s alpha separately for the sample without straightliners and straightliners only. Subsequently, the correlation between summed scores representing the six social media domains (I-VI) were estimated separately for the sample without straightliners and the straightliners only, using Spearman’s rank correlation. Finally, gender-stratified linear regression models were estimated between each social media domain and symptoms of anxiety and symptoms of depression for the full sample, the sample without the straightliners, and straightliners only. Differences between the three samples were assessed by testing whether the unstandardized estimated associations differed significantly across pairs of linear regression models (i.e. pairwise comparisons): sample without straightliners vs. straightliners only and full sample vs. sample without straightliners. We employed robust standard errors to account for potential heteroscedasticity (heteroskedasticity-consistent standard errors), ensuring the validity of our comparisons. In the event of significantly different unstandardized estimates, the standardized β were computed along with the relative difference between them (Δ% = ((β₂ - β₁)/β₁) * 100). All analyses were carried out using R^35^ and RStudio^36^. Identification of straightlining was done by using the package ‘careless’^37^. The package ‘lmtest’^38^ were used compute robust coefficient tests on pairs of linear models, and calculate the coefficient estimates and their standard errors. The coefficients between the two models were then compared and the statistical significance of the difference in coefficients were assessed using the corresponding p-value. Effect size was estimated using ‘RESI’^39^. Tables were produced with the help of ‘gtsummary’^40^, plots with the help of ‘ggplot2’^41^.

## Results

### Prevalence of straightliners and demographic characteristics

**Fig. 1:**
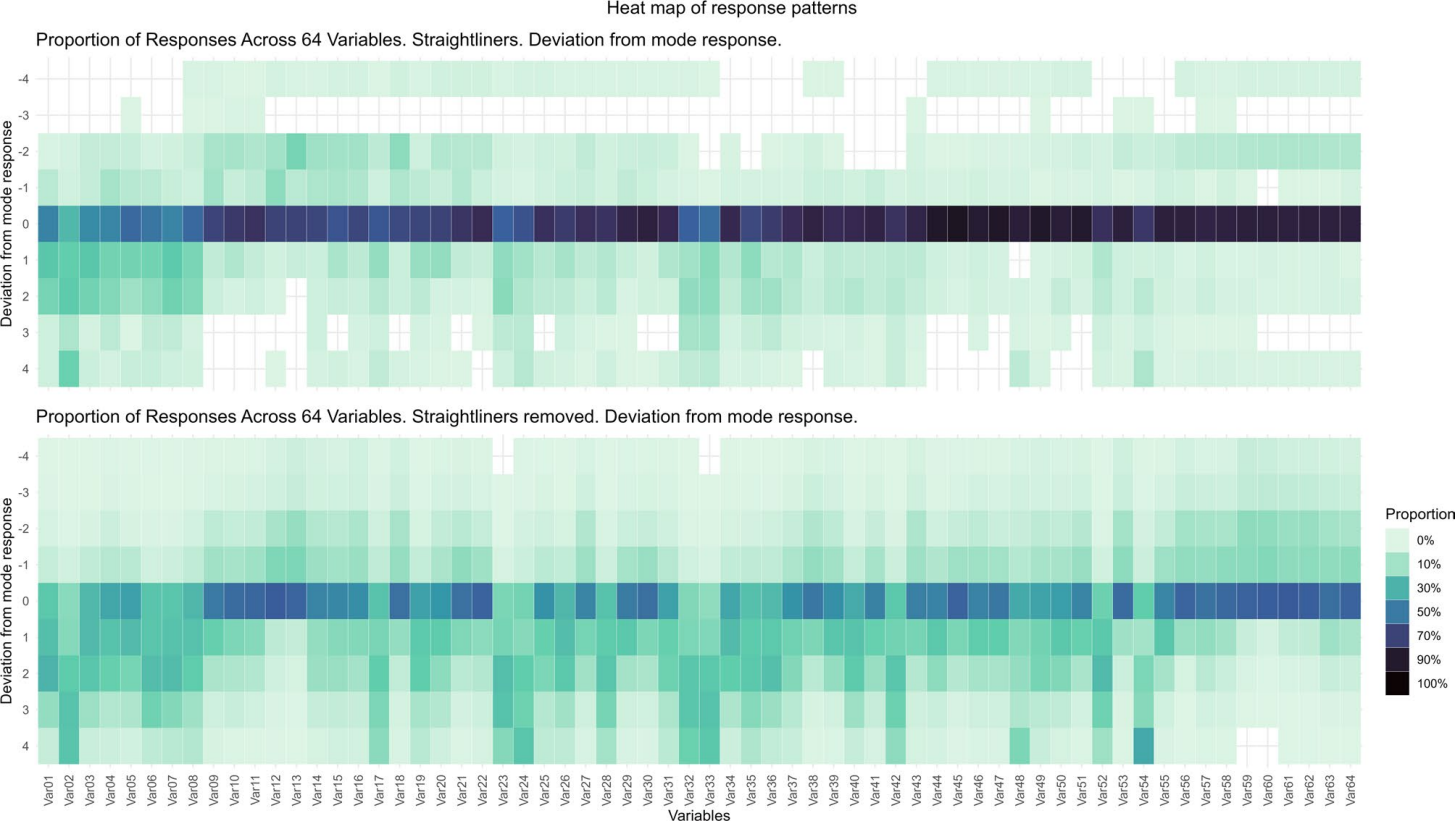
Heatmap of response patterns for straightliners and the remainder of the sample across 64 social media variables. Zero indicates no deviation from each participant’s typical response option (‘mode’) across variables. Darker colours indicate higher proportion, and blank areas represent no observations.

In total, 177 (5.4%) participants were identified as straightliners; 8.6% of boys were straightliners compared to 2.9% of girls (*p* < 0.001). Straightliners exhibited more compressed and less variable response patterns compared to the remainder, with a higher concentration of responses at the extremes (see Supplemental material, Table S1, and figures S2 and S3). There were no differences in the age between straightliners versus the remainder of the sample. The straightliners completed the whole survey slightly faster than the remainder (median ~ 29 min vs. ~ 33 min, *p* < 0.001). The median for the longest identical string of consecutive responses was 24 (38% of the social media items) for straightliners compared to 7 (11% of the social media items) for the remainder of the sample (see Table 1). In Fig. 1, two heatmaps of the response patterns of the sample can be seen. The upper panel is for the straightliners, and the lower panel is for the remainder of the sample across the 64 social media items. The heatmaps clearly illustrate that the straightliners are less likely to deviate from their most typical response across the items, while the remainder of the sample display more variability across items. The heatmap also indicates that there is a tendency to provide longer strings of identical consecutive responses at the end of the social media section of the survey. The average absolute deviation from their most typical response was a median of 0.43 for the straightliners versus 1.08 for the remainder (*p* < 0.001), corresponding to a large robust effect size of 0.41 (95%CI 0.33–0.50).

**Table 1:** Descriptive statistics of included variables across straightliners and remainders.

Characteristic	Overall *N* = 3,285^1^	Straightliners *N* = 177^1^	Remainder *N* = 3,108^1^	*p*-value^2^
Gender				< 0.001
Boys	1,433 (44%)	123 (69%)	1,310 (42%)	
Girls	1,852 (56%)	54 (31%)	1,798 (58%)	
Age				0.9
16	600 (18%)	32 (18%)	568 (18%)	
17	1,573 (48%)	84 (47%)	1,489 (48%)	
18	901 (27%)	52 (29%)	849 (27%)	
19+	211 (6.4%)	9 (5.1%)	202 (6.5%)	
Total time to complete survey (minutes)	32 (27, 40)	29 (22, 38)	33 (27, 40)	< 0.001
Longstring length				< 0.001
Median (Q1, Q3)	7.0 (5.0, 9.0)	24.0 (20.0, 41.0)	7.0 (5.0, 9.0)	
5% Centile, 95% Centile	4.0, 16.0	16.0, 64.0	3.0, 12.0	

^1^n (%); Median (Q1, Q3). ^2^Pearson’s Chi-squared test; Wilcoxon rank sum test.

**Table 2:** Cronbach’s α for sample without straightliners compared with straightliners.

	Cronbach’s Alpha		
Domain	Sample without straightliners	Straightliners only	Difference
Self-presentation	0.87	0.95	0.08
Negative acts and exclusion	0.79	0.99	0.20
Unwanted attention from others	0.82	0.95	0.13
Subjective overuse	0.83	0.90	0.07
Social obligations	0.85	0.91	0.06
Source of concern	0.64	0.90	0.26

**Table 3:** Anxiety (GAD) and depression (SMFQ) regressed on summed scores representing different social media domains. Results from linear regression analyses. Full sample, sample without straightliners and straightliners only. Girls.

	Full sample (*n* = 1,852)			Sample without straightliners (*n* = 1798)				Straightliners only (*n* = 54)	
Variables	Beta (Unstandardized)﻿	95% CI^1^	*p*-value	Beta (Unstandardized)﻿	95% CI^1^	*p*-value	Beta (Unstandardized)﻿	95% CI^1^	*p*-value
Self-presentation^a, b^	1.6	1.3, 1.9	< 0.001	1.5	1.2, 1.8	< 0.001	3.0	1.6, 4.4	< 0.001
Negative acts and exclusion	2.5	2.0, 3.0	< 0.001	2.7	2.1, 3.3	< 0.001	2.2	0.60, 3.9	0.008
Unwanted attention from others	1.6	1.3, 1.9	< 0.001	1.6	1.3, 1.8	< 0.001	2.6	0.96, 4.3	0.003
Subjective overuse^a, b^	0.74	0.46, 1.0	< 0.001	0.69	0.40, 0.97	< 0.001	2.3	0.98, 3.6	0.001
Social obligations^a, b^	1.2	0.90, 1.5	< 0.001	1.1	0.84, 1.4	< 0.001	3.0	1.3, 4.6	< 0.001
Source of concern^a, b^	1.2	0.97, 1.5	< 0.001	1.2	0.91, 1.4	< 0.001	3.0	1.6, 4.3	< 0.001
Symptoms of depression (SMFQ)									
Self-presentation^a, b^	2.2	1.8, 2.5	< 0.001	2.1	1.8, 2.5	< 0.001	3.0	0.01, 6.0	0.049
Negative acts and exclusion	4.0	3.4, 4.6	< 0.001	4.4	3.8, 5.0	< 0.001	2.7	0.21, 5.1	0.034
Unwanted attention from others	2.1	1.7, 2.4	< 0.001	2.0	1.7, 2.4	< 0.001	2.8	0.25, 5.4	0.032
Subjective overuse^a, b^	0.85	0.50, 1.2	< 0.001	0.80	0.44, 1.2	< 0.001	2.3	0.36, 4.2	0.021
Social obligations^a, b^	1.5	1.1, 1.8	< 0.001	1.4	1.0, 1.8	< 0.001	2.8	0.13, 5.4	0.040
Source of concern^a, b^	1.3	0.98, 1.6	< 0.001	1.2	0.91, 1.6	< 0.001	3.2	0.88, 5.5	0.008

^*1*^CI = Confidence Interval for Beta; ^a^Significant difference between sample without straightliners and straightliners only (*p* < 0.05); ^b^Significant difference between full sample and straightliners only (*p* < 0.05).

**Table 4:** Anxiety (GAD) and depression (SMFQ) regressed on summed scores representing different social media domains. Results from linear regression analyses. Full sample, sample without straightliners and straightliners only. Boys.

	Full sample (*n* = 1433)				Sample without straightliners (*n* = 1310)				Straightliners only (*n* = 123)	
Characteristic	Beta (Unstandardized)	95% CI^1^	*p*-value	Beta (Unstandardized)﻿		95% CI^1^	*p*-value	Beta (Unstandardized)﻿	95% CI^1^	*p*-value
Symptoms of anxiety (GAD)										
Self-presentation	1.7	1.3, 2.2	< 0.001	1.8		1.4, 2.3	< 0.001	1.4	0.11, 2.7	0.034
Negative acts and exclusion	1.6	1.1, 2.1	< 0.001	2.0		1.5, 2.5	< 0.001	1.2	0.01, 2.3	0.048
Unwanted attention from others	0.97	0.61, 1.3	< 0.001	0.98		0.63, 1.3	< 0.001	1.1	0.01, 2.2	0.049
Subjective overuse	0.94	0.67, 1.2	< 0.001	0.88		0.60, 1.2	< 0.001	1.3	0.41, 2.2	0.004
Social obligations	1.1	0.82, 1.4	< 0.001	1.0		0.73, 1.4	< 0.001	1.6	0.44, 2.8	0.008
Source of concern	1.3	1.0, 1.6	< 0.001	1.4		1.0, 1.7	< 0.001	1.1	0.10, 2.0	0.031
Symptoms of depression (SMFQ)										
Self-presentation	2.0	1.6, 2.5	< 0.001	2.2		1.7, 2.7	< 0.001	1.4	0.42, 2.4	0.006
Negative acts and exclusion^a^	2.2	1.6, 2.8	< 0.001	3.2		2.5, 3.9	< 0.001	1.2	−0.01, 2.3	0.052
Unwanted attention from others	1.2	0.79, 1.6	< 0.001	1.4		0.94, 1.9	< 0.001	1.1	−0.01, 2.2	0.052
Subjective overuse	1.2	0.92, 1.5	< 0.001	1.1		0.80, 1.4	< 0.001	1.6	0.89, 2.3	< 0.001
Social obligations	1.4	1.1, 1.8	< 0.001	1.4		1.0, 1.8	< 0.001	1.3	0.40, 2.1	0.004
Source of concern	1.4	1.1, 1.7	< 0.001	1.5		1.1, 1.8	< 0.001	0.72	−0.14, 1.6	0.10

^*1*^CI = Confidence Interval for Beta; ^a^Significant difference between sample without straightliners and straightliners only (*p* < 0.05).

### Impact on reliability

**Fig. 2:**
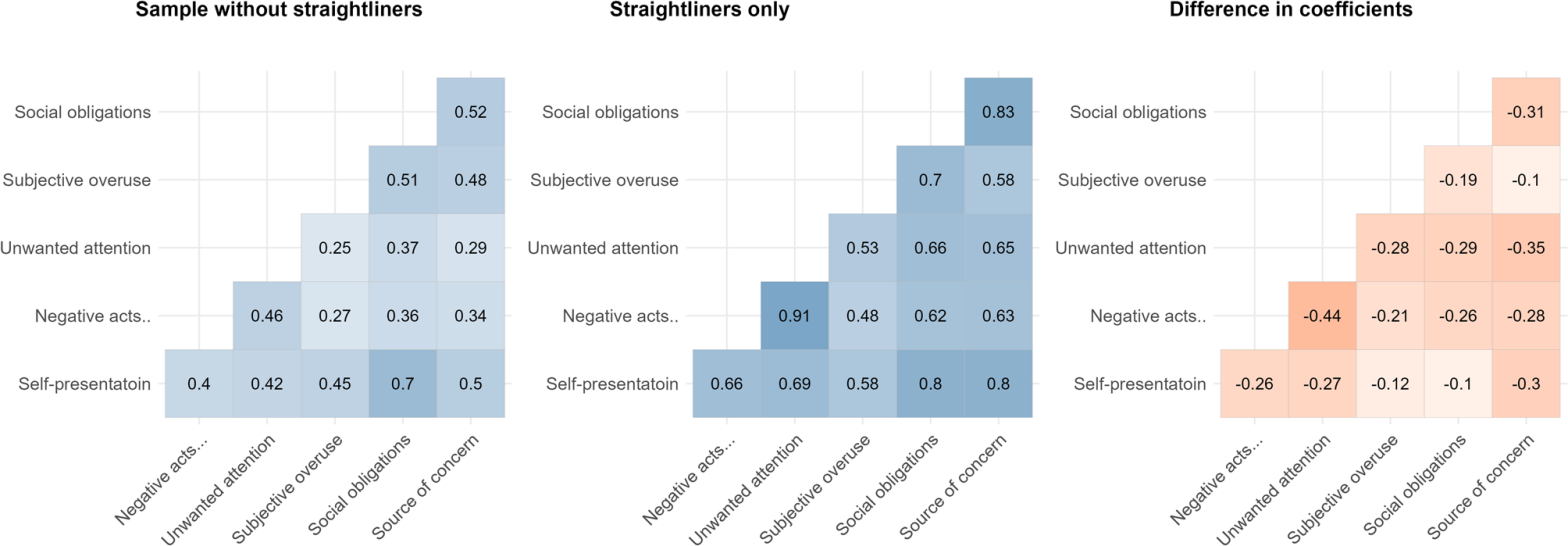
Correlation plots of social media domains. Sample without straigthliners compared with straightliners only. Darker blue colors indicate stronger correlation, and darker red colors indicate larger differences between sample without straightliners and straightliners only.

 For both internal consistency of the domains and the correlations between domains, there was substantial differences between the sample without straightliners and straightliners only (see Table 2; Fig. 2). Both internal consistency of the domains and correlations between domains were, as expected, inflated among straightliners compared to the sample without straightliners. Overall, however, there were only small differences between the full sample and the sample with straightliners removed, both in terms of internal consistency of the domains (mean and maximum difference in Cronbach’s alpha: 0.015 and 0.05) and correlations between domains (mean and maximum difference in correlation coefficient: 0.02 and 0.04).

### Impact on associations with mental health

 For both genders, the point estimates for the associations with symptoms of anxiety and depression differed on face value between the estimates for the full sample and straightliners only, although these differences were not necessarily statistically significant (see Tables 3 and 4). For boys, only one of the estimates was statistically different between the sample without straightliners and straightliners only – the association between negative acts and exclusion and symptoms of depression (β 0.31 vs. β 0.30, relative difference 3%, p-value = 0.003).

For girls, however, four of the associations with symptoms of anxiety were different between the sample without straightliners and straightliners only. Specifically, the estimated associations for the sample without straightliners and straightliners only between self-presentation (β 0.24 vs. β 0.47, relative difference 93%, p-value = 0.034), subjective overuse (β 0.12 vs. β 0.38, relative difference 227%, p-value = 0.017), social obligations (β 0.19 vs. β 0.45, relative difference 140%, p-value = 0.027) and source of concern (β 0.21 vs. β 0.48, relative difference 129%, p-value = 0.009), and symptoms of anxiety were statistically different.

As expected, the associations with symptoms of anxiety and depression were less different between the estimates for the full sample versus the sample where the straightliners were removed for both genders (see Tables 3 and 4). For boys there were no statistically significant differences in the estimates between the full sample and the sample without straightliners (all p-values > 0.05). For girls, however, the estimated associations for the full sample and the sample without straightliners between self-presentation (β 0.25 vs. β 0.47, relative difference 87%), subjective overuse (β 0.13 vs. β 0.38, relative difference 202%), social obligations (β 0.19 vs. β 0.45, relative difference 130%) and source of concern (β 0.22 vs. β 0.48, relative difference 119%), and symptoms of anxiety were statistically different, as they were in the previous comparison (sample without straightliners vs. straightliners).

## Discussion

The present study found that a relatively small percentage of adolescents exhibited straightlining behavior, but with boys being much more likely to do so than girls. Previous studies have also found straightlining to be more prevalent in adolescent boys than girls^8^. Straightliners tended to have very little variability across the 64 social media items compared to others. This is reflected in the apparent higher reliability and correlation between domains among straightliners only compared to the sample without straightliners. Despite this, the estimated reliability and correlation between social media domains remained almost entirely unaffected when straightliners were excluded, probably a reflection of the relatively low prevalence of straightliners in the present sample.

For the associations between domains of social media use and mental health symptoms, there were some notable differences in the estimates across comparisons. The most consistent differences were for girls and four domains of social media in relation to symptoms of anxiety. For both comparisons between the sample without straightliners versus straightliners only, and between the full sample and straightliners only, the estimates were substantially inflated for straightliners only for both unstandardized and standardized estimates. This inflation may be attributed to artificially inflated internal reliability within the domains covered among straightliners, as their consistent responses could create stronger apparent relationships between variables, potentially distorting the overall findings of the analysis.

For boys, our analysis revealed an intriguing pattern in the relationship between negative acts and exclusion, and symptoms of depression. In the sample without straightliners, we found a stronger absolute relationship (b = 3.2) compared to the straightliners only sample (b = 1.2). However, when these coefficients were standardized, they showed remarkable similarity (β ≈ 0.3 for both samples). This discrepancy between unstandardized and standardized coefficients likely reflects fundamental differences in response patterns and variability between the two groups. The straightliners only, characterized by consistent responses across items, appears to use a compressed range of the scale. This compression reduces the absolute magnitude of the relationship (lower unstandardized beta) but maintains a similar relative strength when standardized. The similarity in standardized coefficients suggests that relative to the variability within each group, the estimated strength of the relationship between them is consistent. However, the absolute impact (as indicated by unstandardized coefficients) is notably stronger in the sample without straightliners. These findings highlight the importance of considering both unstandardized and standardized coefficients, especially when dealing with groups that have distinctly different response patterns. It also underscores the need for careful interpretation of results when analyzing data that includes straightliners responses, even when the relative population of straightliners is limited.

Compared to previous studies, the prevalence of straightlining was in the lower end of the spectrum among adolescents^8^, but we did reiterate previous findings of a substantial gender difference. Overall, the impact of straightlining behaviour in our sample was quite minimal, and it is doubtful that the overall conclusions from analytical epidemiological methods such as estimates of associations would differ substantially with or without the straightliners included. Importantly, this is mostly due to the limited number of straightliners identified in our sample, as can be seen by the relatively large discrepancy when comparing the other estimates with the estimates from the sample with straightliners only.

Although straightlining can be a particularly pronounced challenge in online surveys^1^, especially when they are long, we can only speculate that the topic of the present survey (‘social media and wellbeing’) was seen as particularly relevant and interesting for the adolescents who participated, given the central role social media plays in their daily lives and the limited attention this topic typically receives from the adults in their lives^23,42^. Thus, this could have reduced the likelihood of careless responding. It should also be noted that the survey was introduced in a school-setting which may have increased the legitimacy of the survey, which may have been beneficial vis-à-vis response efforts.

### Relevance and potential mitigation

The finding that straightlining behavior is more prevalent among adolescent boys and leads to inflated estimates in social media use and mental health symptom associations highlights the need for careful consideration of response patterns in survey design, especially when using similar response options across long sections of the survey. By identifying and accounting for straightliners, researchers can potentially improve the accuracy and reliability of their findings, ensuring that the data more accurately reflects true associations. Because satisficing, especially straightlining, can introduce systematic errors and biases into survey data, which may undermine data quality and interpretability, some researchers have investigated possible measures to mitigate these issues. However, efforts to reduce or exclude such responses do not necessarily lead to more accurate data. For example, while attention checks can help identify inattentive respondents, removing them from the dataset may increase bias and reduce the representativeness of the sample, particularly if attentiveness is linked to the behavior or attitudes of interest^43^. Furthermore, although straightlining and speeding are often seen as indicators of careless responding, quick responses can sometimes reflect well established attitudes rather than low engagement^5^. Thus, quick responses sometimes capture intuitive or well-formed attitudes just as accurately as slow responses. One study found that up to 40% of participants showed straightlining behaviors, but only about 2% did so in ways that seemed implausible^5^. Other findings support this idea by differentiating between ‘valid’ and ‘invalid’ straightlining, suggesting that certain responses may indeed represent stable, genuine attitudes^44^. This highlights the complexity of addressing satisficing without inadvertently compromising data robustness.

### Strengths and limitations

The present study has several strengths. Firstly, this is the first study to investigate straightlining in relation to questions on social media among adolescents. This is important to investigate since vast research efforts are focused on this topic. In our sample, the prevalence of straightlining was limited, and we believe that this may be at least partly because social media is an engaging topic for adolescents, as well as a topic they have a lot of experience with. Secondly, we were able to use two different mental health outcomes as comparators when investigating the potential impact of straightlining. Thirdly, the relatively large sample size allowed for a gender-specific investigation of the potential impact of straightlining. Finally, we employed a heterogeneous set of statements related to aspects of social media use where we do not expect the inter-item correlations to be high across all of the included statements. This is especially beneficial when we want to identify a systematic tendency to respond similarly across different topical areas, and are not only interested in perfect straightliners^45^.

Some notable limitations also need to be addressed. It is possible that the participants took the survey more seriously than they would have if the survey was unrelated to activities at school. Thus, the present findings may not necessarily generalize to other surveys involving adolescents. On the other hand, some participants may have felt compelled to complete the survey because it was conveyed in a school setting. Consequently, they may have been minimally motivated to complete the survey, resulting in more straightlining behaviour. Under other conditions, these participants may have declined to participate altogether. One study showed that reluctant participants had lower response quality compared to cooperative participants, however, this effect disappeared after controlling for cognitive ability^46^. Also, a key limitation of this study is the substantial imbalance in group sizes between the straightlining group and the remainder, which reduces statistical power for detecting differences, especially for girls. The small number of observations in the straightlining group substantially reduces statistical power to detect group differences, thereby increasing the risk of Type II error and potentially accounting for the absence of statistically significant findings. While robust standard errors were used to account for heteroscedasticity, the increased uncertainty in estimates from the smaller group should be borne in mind when interpreting the findings.

Also, our definition of straightlining, which was based on identifying the statistical outliers, needs to be critically considered. Perfect straightlining is present when all responses are identical across items in a questionnaire. Using this definition would identify only seven girls and seven boys in our sample as straightliners. In the present study we were also interested in identifying partial straightlining, as we were interested in the tendency towards identical responses across 64 items covering different domains and aspects of social media use. This more ‘lenient’ definition of straightlining is recommended when perfect straightlining is very rare (< 5%^2^), and we believe the group identified by our approach is worth investigating, as they display remarkably similar intraindividual response patterns across long sections of the survey part covering social media use (as presented in Fig. 1). We think our definition is more appropriate when trying to identify straightlining across larger numbers of variables than what is usual^2^, especially when the variables are not supposed to gauge the same underlying construct. Finally, we are not able to discern between valid and invalid straightlining – and it is possible that our definition of straightlining also includes some valid response patterns (‘false positives’) that should not have been included among the straightliners^44^. Importantly, our operationalization of straightlining, based on statistical outliers in identical response strings, does not capture contextual inconsistencies. Future research should incorporate qualitative aspects to further explore straightlining behaviour among adolescents. Overall, by using our definition of straightlining, we may have both underestimated and overestimated the impact but there are no infallible way to identify straightlining as a consequence of careless responding^2^.

## Conclusions

In a sample of older adolescents, the present study investigated the prevalence and impact of straightlining across 64 items measuring different aspects of social media use. Although boys were more likely to exhibit straightlining than girls, the overall prevalence was low in the present sample. This also limited the impact of straightlining in terms of analytical epidemiology and likely also the impact on the conclusions drawn. The discrepancy between those categorized as straightliners and the remainder of the sample was, however, large for all parameters investigated, and this may pose a risk in other samples where straightlining is more prevalent. Careless responding, such as straightlining, continue to pose a risk for biased estimates and erroneous interpretations in survey-based epidemiology but may be less pronounced if the topic of research is deemed particularly relevant or interesting for the participants and is potentially dependent on the context of the survey.

## Supplementary Information

Below is the link to the electronic supplementary material.

Supplementary Material 1

## Data Availability

The datasets analysed during the current study are not publicly available, as they contain sensitive information, and the ethical approval of the study does not include this option. Requests to access these datasets should be directed to JCS, jens.christoffer.skogen@fhi.no.
