# Supplementary material for: Straightlining prevalence across domains of social media use and impact on internal consistency and mental health associations in the LifeOnSoMe study
Source: Sci Rep. 2025 Aug 7;15:28990. doi: 10.1038/s41598-025-14276-6 (PMC12332011; doi:10.1038/s41598-025-14276-6)
Supplement: Supplementary file 1 — Supplementary Material 1 [file 41598_2025_14276_MOESM1_ESM.docx]

**Supplementary material:** Straightlining prevalence across domains of social media use and impact on internal consistency and mental health associations in the LifeOnSoMe study.

**Introductory text and introductory questions.**

*You will now be presented with some questions and statements about your use of social media. Social media is a collective term for all two-way communication that takes place over the internet, websites, and apps (including online gaming). All the following items apply to social media, although this is not specified in every question.*

| **Do you use social media?** |
| --- |
| **Answer options:** |
| Yes |
| No, never |

| **How often do you use social media?** |
| --- |
| **Answer options:** |
| Almost never |
| Several times a month, but less frequently than every week |
| 1-2 times a week |
| 3-4 times a week |
| 5-6 times a week |
| Every day |
| Several times a day |
| Almost all the time |

| **The days you use social media, approximately how much time do you spend per day?** |
| --- |
| **Answer options:** |
| Less than 30 minutes |
| 30 minutes-1 hour |
| >1-2 hours |
| >2-3 hours |
| >3-4 hours |
| >4-5 hours |
| More than 5 hours |

| **What social media platforms do you use? Multiple responses are possible** |
| --- |
| **Answer options:** |
| Facebook |
| Instagram |
| Snapchat |
| LinkedIn |
| Twitter |
| YouTube |
| Pinterest |
| Messenger |
| WhatsApp |
| Yodel |
| TikTok |
| Discord |
| Twitch |
| Spond |
| Anonymous apps (e.g. Ask, Sarahah) |
| Dating apps (e.g. Tinder, Match, Grindr, Yubo) |
| Houseparty |
| Reddit |
| Other |

**List of social media activities.**

**Question: When using social media, how often do you do the following?**

**Answer options: Several times a day, daily, weekly, less than weekly, never**

1. Use social media related to schoolwork
2. I use social media during school hours (for things other than schoolwork)
3. Write posts, post links or comments on issues or debates related to news, society, culture, or politics
4. Update myself on what is happening among friends, groups I am part of, or about cultural events
5. News update
6. Learn new things (e.g. on YouTube)
7. Post status updates / stories or photos / video of myself / friends
8. Chat with friends one-on-one
9. Chat with friends in groups
10. Share photos / memes / videos with friends / family
11. Look at photos / videos friends and others have shared (including celebrities and the like)
12. Exchange music, movies, or other things I am interested in with like-minded people
13. Communicate with others through anonymous apps / features
14. Use dating apps like Tinder or something similar
15. Communicate with others in connection with gaming (e.g. through Discord)
16. Watch and comment on others' gaming (e.g. on Twitch)

**Different aspects of social media use.**

**Question: To what extent are the following statements true for you?**

**Answer options: Not at all, A little, Somewhat/partly, A lot, Very much**

1. Social media takes away focus from more important things
2. My mobile is always available, even when I have gone to bed
3. I am addicted to social media
4. My parents/guardians think I spend too much time on social media
5. I fear I might miss out on something if I am not on social media
6. Social media gives me a sense of control or overview of what is going on
7. I spend too much time on social media
8. I want to reduce the amount of time I spend on social media
9. I spend a lot of time and energy on what I post on social media
10. It is important for me to get many likes and/or comments on what I post on social media
11. It is important for me to have many followers on social media
12. I delete what I post on social media if it does not get enough likes or comments
13. I retouch photos of myself to look better before posting them on social media
14. It's easier to be myself on social media
15. I have several profiles or usernames on the same platform
16. I am concerned with making sure that what I do online cannot be linked to my name
17. I'm careful about what I post because I consider how it might affect me in the future
18. There are pictures / video / other of me on the internet that I do not want others to see
19. I feel that I must like and/or comment on what friends post on social media
20. I feel that I must respond to all messages, "streaks" and similar things I receive
21. If I do not respond, like or comment, then it can have negative consequences
22. If friends do not like or comment on what I post on social media, I start thinking something is wrong
23. I pick up the phone as soon as I have nothing to do
24. I turn the phone on silent / "do not disturb" in situations where I have to concentrate / get something done
25. I use my mobile phone to distract myself from painful thoughts or feelings
26. I pick up the phone as soon as it becomes quiet in a conversation or a situation is embarrassing or uncomfortable
27. I prefer to talk to people through social media rather than in real life
28. If I have a bad day, I use social media mostly as entertainment and am less socially active
29. Social media is a stress factor in my life
30. There is so much happening on social media that I often feel overwhelmed
31. I wish we could learn more about how social media affects us
32. I keep in touch with friends and family through social media that I do not otherwise see in everyday life
33. I have ongoing contact with friends throughout the day on social media
34. If I do not participate on social media, I will fall behind
35. Through social media, I get in touch with others who have the same interests as me
36. I get a lot of positive attention through social media
37. It's easier for me to talk about difficult things through social media
38. I often end up in disputes or misunderstandings based on misinterpretations of what I have sent / shared
39. I find it difficult to interpret what people write / send / post
40. I follow closely what my friends/girlfriend/boyfriend/ family does through social media (for example stories, Snap map…)
41. Sometimes I feel like I am being monitored on social media (because what I do/where I am/who I am with is visible)
42. I find that people behave worse on social media if they are anonymous
43. I have done / written things on social media that I would never do in real life
44. What others post (photos/status updates/stories) makes me feel less content with myself and my own life
45. The response I get to what I post (photos/status updates/stories) impacts how I feel
46. I often find that my friends have group conversations that I do not participate in
47. I experience that not being included in a private story or something similar makes me feel down
48. It does not matter to me how many likes or comments I get on social media
49. I like to have periods where I do not have access to social media
50. My parents show interest in what I do on social media
51. I often experience that my parents / guardians are so busy with their mobile phones that I can´t get their attention

**Question: How often does the following happen/do you do the following:**

**Answer options: "Never, Seldom, Sometimes, Often, Very often**

1. I go to bed later than I should because I'm on social media in the evening
2. I get woken up by notifications on my mobile phone after falling asleep at night
3. I turn the phone on silent / "do not disturb" when I go to sleep
4. I get contacted/get unwanted attention from strangers on social media
5. Others share photos/videos of me against my will
6. I receive nude photos or sexualized content from others without asking for it
7. I am asked to send nude photos or sexualized content of myself to others
8. I spread pictures of others without the person being aware of it
9. I get negative/rude comments on what I post
10. I receive unpleasant or hurtful messages through social media
11. Others say/post bad things about me on social media
12. I participate in gossiping or talking badly about others on social media (e.g. in group chat)
13. I feel excluded from groups/group chats on social media

**Colour codes for social media domains investigated.**

| I | Self-presentation (Social comparison and self-presentation) |
| --- | --- |
| II | Negative acts and exclusion |
| III | Unwanted attention from others |
| IV | Subjective overuse |
| V | Social obligations |
| VI | Source of concern |

| 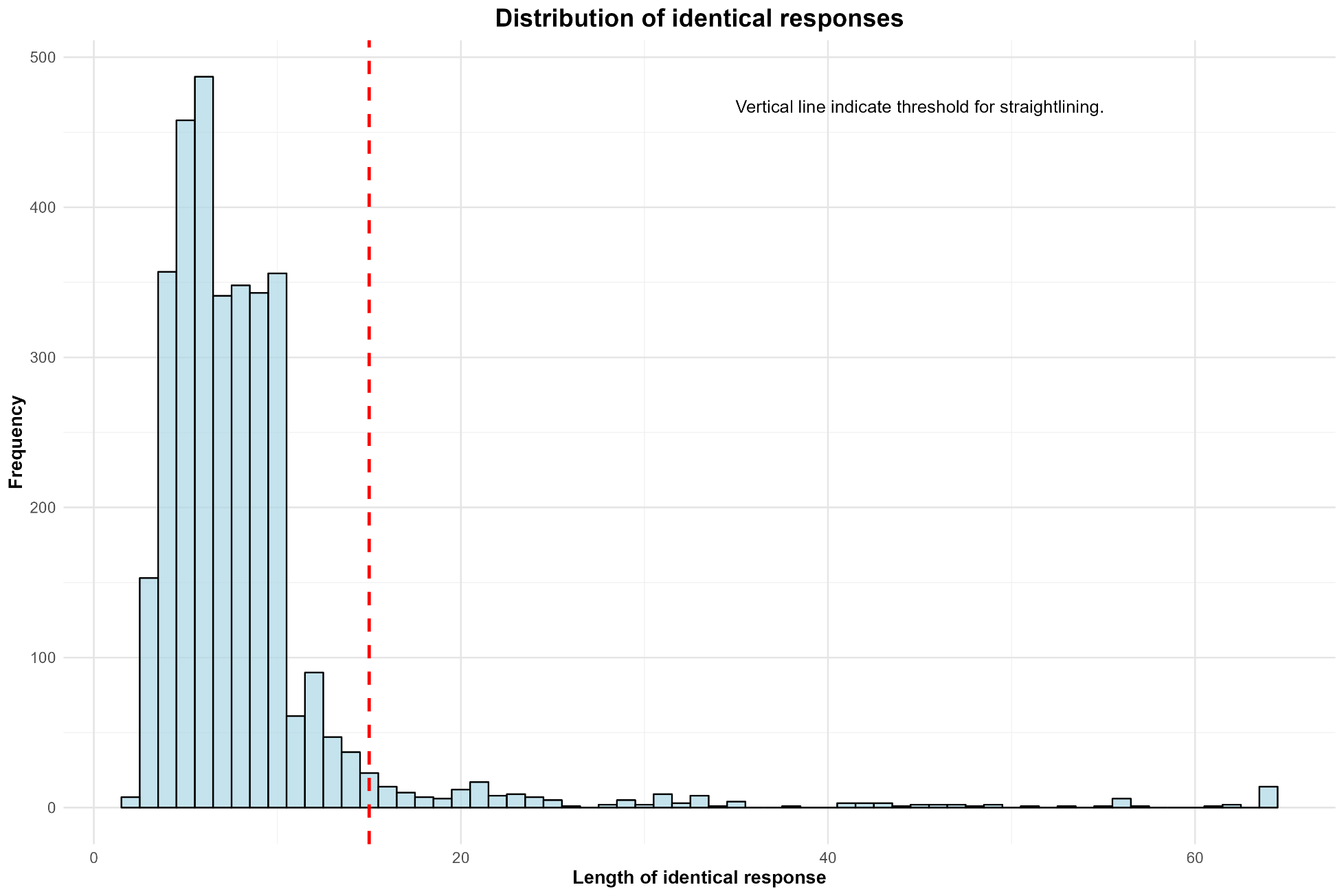 |
| --- |

Figure S1: Distribution of identical responses. Red dotted vertical line indicate threshold for straightlining.

Table S1: Descriptive statistics of social media variables across straightliners and remainders.

| \| **SOME-variables** \| **Straightliners**  N = 177^1^ \| **Remainder**  N = 3,108^1^ \| **p-value**^2^ \| \| --- \| --- \| --- \| --- \| \| 1. Social media takes away focus from more important things \| 2.73 (1.18) \| 3.17 (0.99) \| <0.001 \| \| 2. My mobile is always available, even when I have gone to bed \| 3.63 (1.31) \| 4.07 (1.08) \| <0.001 \| \| 3. I am addicted to social media \| 2.69 (1.26) \| 3.01 (1.14) \| <0.001 \| \| 4. My parents/guardians think I spend too much time on social media \| 2.59 (1.24) \| 2.67 (1.19) \| 0.4 \| \| 5. I fear I might miss out on something if I am not on social media \| 2.46 (1.22) \| 2.71 (1.22) \| 0.006 \| \| 6. Social media gives me a sense of control or overview of what is going on \| 2.72 (1.26) \| 3.34 (1.14) \| <0.001 \| \| 7. I spend too much time on social media \| 2.81 (1.30) \| 3.30 (1.13) \| <0.001 \| \| 8. I want to reduce the amount of time I spend on social media \| 2.50 (1.25) \| 3.00 (1.26) \| <0.001 \| \| 9. I spend a lot of time and energy on what I post on social media \| 1.83 (1.06) \| 2.11 (1.15) \| <0.001 \| \| 10. It is important for me to get many likes and/or comments on what I post on social media \| 1.87 (1.13) \| 2.10 (1.19) \| 0.006 \| \| 11. It is important for me to have many followers on social media \| 1.89 (1.08) \| 1.98 (1.10) \| 0.2 \| \| 12. I delete what I post on social media if it does not get enough likes or comments \| 1.72 (1.09) \| 1.53 (0.91) \| 0.029 \| \| 13. I retouch photos of myself to look better before posting them on social media \| 1.65 (1.00) \| 1.33 (0.73) \| <0.001 \| \| 14. It's easier to be myself on social media \| 1.95 (1.19) \| 2.00 (1.12) \| 0.3 \| \| 15. I have several profiles or usernames on the same platform \| 1.95 (1.17) \| 2.13 (1.25) \| 0.058 \| \| 16. I am concerned with making sure that what I do online cannot be linked to my name \| 1.93 (1.14) \| 1.97 (1.09) \| 0.4 \| \| 17. I'm careful about what I post because I consider how it might affect me in the future \| 2.46 (1.44) \| 3.33 (1.28) \| <0.001 \| \| 18. There are pictures / video / other of me on the internet that I do not want others to see \| 1.79 (1.14) \| 1.70 (0.99) \| 0.7 \| \| 19. I feel that I must like and/or comment on what friends post on social media \| 2.24 (1.33) \| 2.93 (1.41) \| <0.001 \| \| 20. I feel that I must respond to all messages, 'streaks' and similar things I receive \| 2.00 (1.18) \| 2.42 (1.27) \| <0.001 \| \| 21. If I do not respond, like or comment, then it can have negative consequences \| 1.87 (1.11) \| 1.89 (1.03) \| 0.4 \| \| 22. If friends do not like or comment on what I post on social media, I start thinking something is wrong \| 1.88 (1.14) \| 2.05 (1.22) \| 0.052 \| \| 23. I pick up the phone as soon as I have nothing to do \| 2.65 (1.38) \| 3.87 (1.05) \| <0.001 \| \| 24. I turn the phone on silent / 'do not disturb' in situations where I have to concentrate / get something done \| 2.67 (1.40) \| 3.80 (1.24) \| <0.001 \| \| 25. I use my mobile phone to distract myself from painful thoughts or feelings \| 2.19 (1.30) \| 2.68 (1.40) \| <0.001 \| \| 26. I pick up the phone as soon as it becomes quiet in a conversation or a situation is embarrassing or uncomfortable \| 2.30 (1.27) \| 3.03 (1.22) \| <0.001 \| \| 27. I prefer to talk to people through social media rather than in real life \| 2.06 (1.18) \| 1.99 (1.05) \| 0.7 \| \| 28. If I have a bad day, I use social media mostly as entertainment and am less socially active \| 2.29 (1.31) \| 3.40 (1.22) \| <0.001 \| \| 29. Social media is a stress factor in my life \| 1.99 (1.16) \| 2.20 (1.09) \| 0.005 \| \| 30. There is so much happening on social media that I often feel overwhelmed \| 2.00 (1.15) \| 2.05 (1.11) \| 0.4 \| \| 31. I wish we could learn more about how social media affects us \| 2.15 (1.23) \| 2.76 (1.29) \| <0.001 \| \| 32. I keep in touch with friends and family through social media that I do not otherwise see in everyday life \| 2.84 (1.28) \| 3.90 (1.03) \| <0.001 \| \| 33. I have ongoing contact with friends throughout the day on social media \| 2.99 (1.28) \| 4.08 (1.02) \| <0.001 \| \| 34. If I do not participate on social media, I will fall behind \| 2.28 (1.24) \| 2.82 (1.18) \| <0.001 \| \| 35. Through social media, I get in touch with others who have the same interests as me \| 2.57 (1.22) \| 3.18 (1.18) \| <0.001 \| \| 36. I get a lot of positive attention through social media \| 2.52 (1.22) \| 3.02 (1.12) \| <0.001 \| \| 37. It's easier for me to talk about difficult things through social media \| 2.32 (1.21) \| 2.49 (1.29) \| 0.12 \| \| 38. I often end up in disputes or misunderstandings based on misinterpretations of what I have sent / shared \| 2.11 (1.19) \| 1.79 (0.94) \| 0.001 \| \| 39. I find it difficult to interpret what people write / send / post \| 2.27 (1.19) \| 2.13 (0.94) \| 0.2 \| \| 40. I follow closely what my friends/girlfriend/boyfriend/ family does through social media (for example stories, Snap map…) \| 2.32 (1.25) \| 2.94 (1.26) \| <0.001 \| \| 41. Sometimes I feel like I am being monitored on social media (because what I do/where I am/who I am with is visible) \| 2.20 (1.20) \| 2.25 (1.14) \| 0.4 \| \| 42. I find that people behave worse on social media if they are anonymous \| 2.50 (1.32) \| 3.56 (1.25) \| <0.001 \| \| 43. I have done / written things on social media that I would never do in real life \| 2.18 (1.18) \| 1.91 (1.08) \| 0.003 \| \| 44. What others post (photos/status updates/stories) makes me feel less content with myself and my own life \| 2.05 (1.14) \| 2.39 (1.21) \| <0.001 \| \| 45. The response I get to what I post (photos/status updates/stories) impacts how I feel \| 2.02 (1.11) \| 1.99 (1.05) \| >0.9 \| \| 46. I often find that my friends have group conversations that I do not participate in \| 2.07 (1.11) \| 2.08 (1.05) \| 0.9 \| \| 47. I experience that not being included in a private story or something similar makes me feel down \| 2.02 (1.11) \| 2.15 (1.12) \| 0.13 \| \| 48. It does not matter to me how many likes or comments I get on social media \| 2.26 (1.29) \| 2.90 (1.40) \| <0.001 \| \| 49. I like to have periods where I do not have access to social media \| 2.05 (1.14) \| 2.39 (1.22) \| <0.001 \| \| 50. My parents show interest in what I do on social media \| 2.09 (1.12) \| 2.34 (1.06) \| 0.002 \| \| 51. I often experience that my parents / guardians are so busy with their mobile phones that I can't get their attention \| 1.97 (1.08) \| 1.96 (1.00) \| 0.9 \| \| 52. I go to bed later than I should because I'm on social media in the evening \| 2.37 (1.21) \| 3.57 (1.13) \| <0.001 \| \| 53. I get woken up by notifications on my mobile phone after falling asleep at night \| 2.11 (1.19) \| 1.54 (0.88) \| <0.001 \| \| 54. I turn the phone on silent / 'do not disturb' when I go to sleep \| 2.53 (1.38) \| 3.96 (1.50) \| <0.001 \| \| 55. I get contacted/get unwanted attention from strangers on social media \| 2.10 (1.19) \| 2.11 (1.03) \| 0.5 \| \| 56. Others share photos/videos of me against my will \| 1.96 (1.14) \| 1.50 (0.71) \| <0.001 \| \| 57. I receive nude photos or sexualized content from others without asking for it \| 2.04 (1.17) \| 1.78 (1.02) \| 0.007 \| \| 58. I am asked to send nude photos or sexualized content of myself to others \| 1.98 (1.16) \| 1.66 (0.98) \| <0.001 \| \| 59. I spread pictures of others without the person being aware of it \| 1.94 (1.15) \| 1.20 (0.54) \| <0.001 \| \| 60. I get negative/rude comments on what I post \| 1.92 (1.16) \| 1.17 (0.48) \| <0.001 \| \| 61. I receive unpleasant or hurtful messages through social media \| 1.90 (1.14) \| 1.32 (0.66) \| <0.001 \| \| 62. Others say/post bad things about me on social media \| 1.89 (1.15) \| 1.33 (0.67) \| <0.001 \| \| 63. I participate in gossiping or talking badly about others on social media (e.g. in group chat) \| 1.92 (1.15) \| 1.53 (0.82) \| <0.001 \| \| 64. I feel excluded from groups/group chats on social media \| 1.90 (1.13) \| 1.54 (0.84) \| <0.001 \| \| ^1^Mean (SD) \| \| \| \| \| ^2^Wilcoxon rank sum test \| \| \| \| |
| --- | --- | --- | --- | --- | --- | --- | --- | --- | --- | --- | --- | --- | --- | --- | --- | --- | --- | --- | --- | --- | --- | --- | --- | --- | --- | --- | --- | --- | --- | --- | --- | --- | --- | --- | --- | --- | --- | --- | --- | --- | --- | --- | --- | --- | --- | --- | --- | --- | --- | --- | --- | --- | --- | --- | --- | --- | --- | --- | --- | --- | --- | --- | --- | --- | --- | --- | --- | --- | --- | --- | --- | --- | --- | --- | --- | --- | --- | --- | --- | --- | --- | --- | --- | --- | --- | --- | --- | --- | --- | --- | --- | --- | --- | --- | --- | --- | --- | --- | --- | --- | --- | --- | --- | --- | --- | --- | --- | --- | --- | --- | --- | --- | --- | --- | --- | --- | --- | --- | --- | --- | --- | --- | --- | --- | --- | --- | --- | --- | --- | --- | --- | --- | --- | --- | --- | --- | --- | --- | --- | --- | --- | --- | --- | --- | --- | --- | --- | --- | --- | --- | --- | --- | --- | --- | --- | --- | --- | --- | --- | --- | --- | --- | --- | --- | --- | --- | --- | --- | --- | --- | --- | --- | --- | --- | --- | --- | --- | --- | --- | --- | --- | --- | --- | --- | --- | --- | --- | --- | --- | --- | --- | --- | --- | --- | --- | --- | --- | --- | --- | --- | --- | --- | --- | --- | --- | --- | --- | --- | --- | --- | --- | --- | --- | --- | --- | --- | --- | --- | --- | --- | --- | --- | --- | --- | --- | --- | --- | --- | --- | --- | --- | --- | --- | --- | --- | --- | --- | --- | --- | --- | --- | --- | --- | --- | --- | --- | --- | --- | --- | --- | --- | --- | --- | --- | --- | --- | --- | --- | --- | --- | --- | --- | --- | --- | --- | --- | --- | --- |


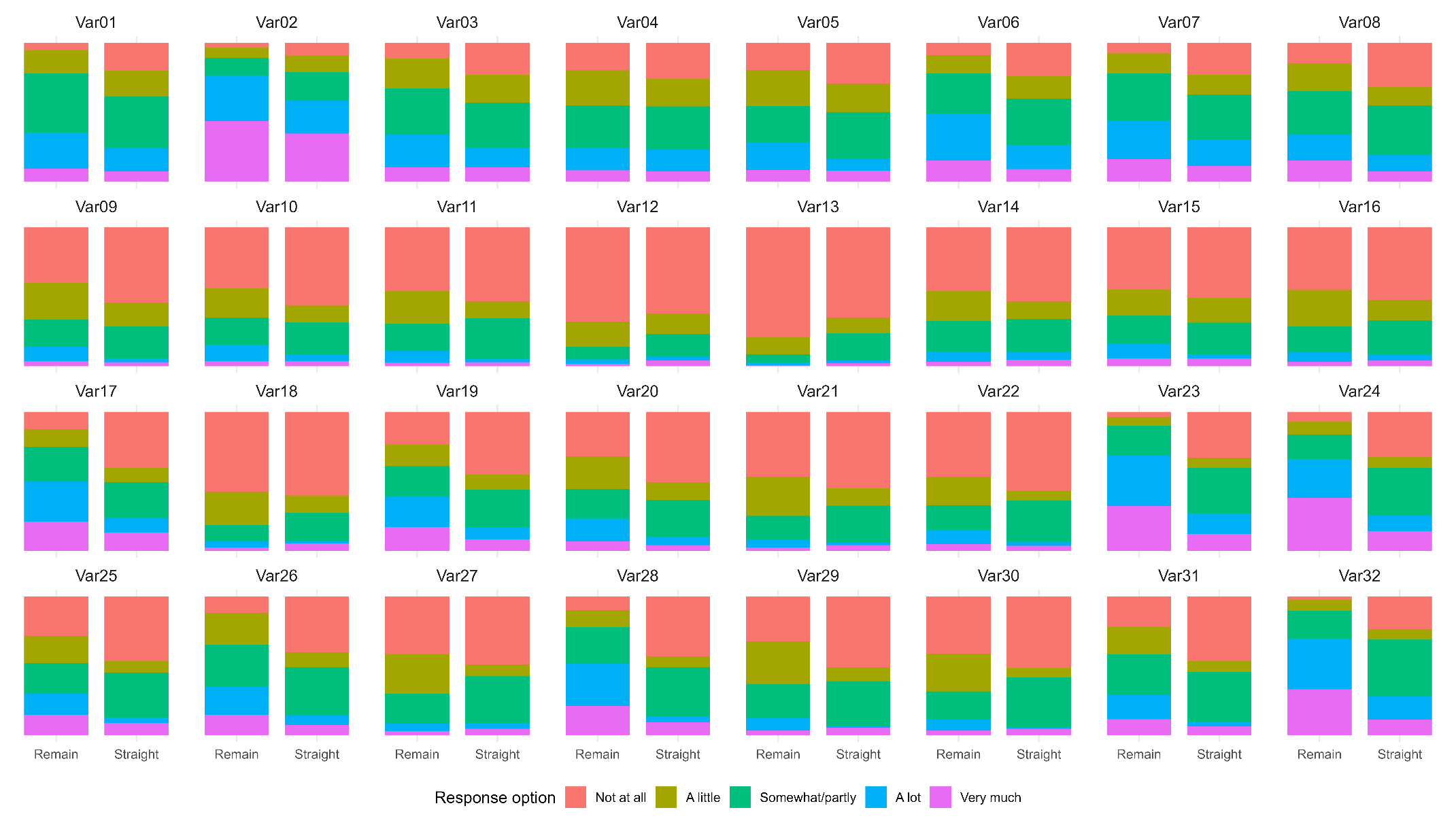


Figure S2: Distribution of SOME-variables 1–32. 'Straight' indicates straightliners and 'Remain' indicates the remainder.


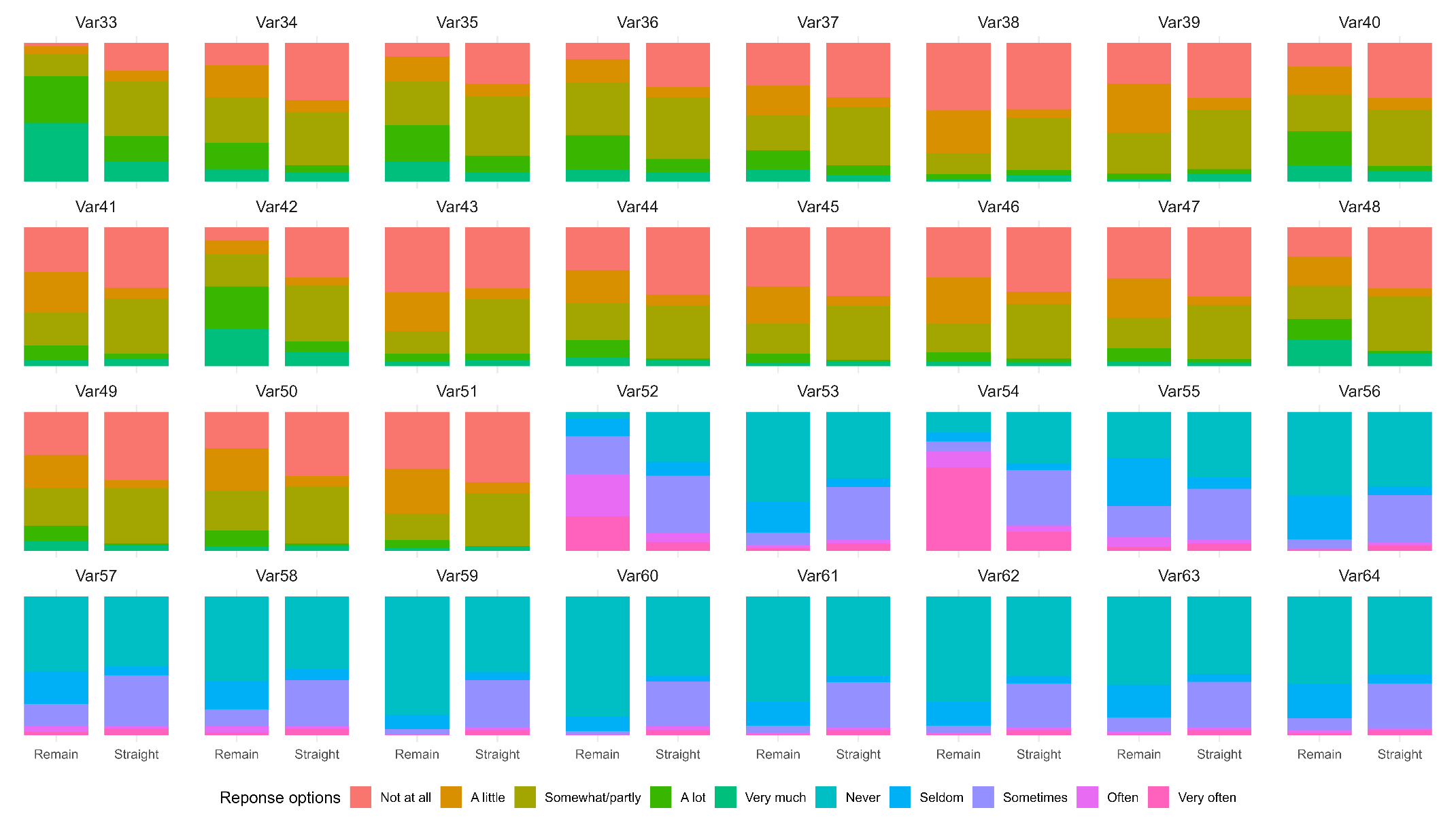


Figure S3: Distribution of SOME-variables 33–64. 'Straight' indicates straightliners and 'Remain' indicates the remainder.

Note: SOME-variables 33-51 have "Not at all", "A little", "Somewhat/partly", "A lot", "Very much" as the response options while 52-64 have "Never", "Seldom", "Sometimes", "Often", "Very often" as the response options.
